# Cytotoxic and proinflammatory effects of PVP-coated silver nanoparticles after intratracheal instillation in rats

**DOI:** 10.3762/bjnano.4.105

**Published:** 2013-12-19

**Authors:** Nadine Haberl, Stephanie Hirn, Alexander Wenk, Jörg Diendorf, Matthias Epple, Blair D Johnston, Fritz Krombach, Wolfgang G Kreyling, Carsten Schleh

**Affiliations:** 1 Institute of Lung Biology and Disease, Helmholtz Center Munich, Neuherberg/Munich, Germany; 2 Current address: Walter Brendel Centre of Experimental Medicine, Ludwig-Maximilians-Universität München, Marchioninistr. 15, 81377 Munich, Germany, Phone: +49 89 2180 76540, Fax: +4949 89 2180 76532; 3 Inorganic Chemistry and Center of Nanointegration Duisburg-Essen, University of Duisburg-Essen, Essen, Germany; 4 Walter Brendel Centre of Experimental Medicine, Ludwig-Maximilians-Universität München, Munich, Germany; 5 Current address: Institute of Epidemiology 2, Helmholtz Center Munich, Neuherberg/Munich, Germany; 6 Current address: Berufsgenossenschaft Holz und Metall, Am Knie 8, 81241 München, Germany

**Keywords:** cytotoxicity, inflammation, pulmonary toxicity, silver nanoparticles

## Abstract

Silver nanoparticles (AgNP) are among the most promising nanomaterials, and their usage in medical applications and consumer products is growing rapidly. To evaluate possible adverse health effects, especially to the lungs, the current study focused on the cytotoxic and proinflammatory effects of AgNP after the intratracheal instillation in rats. Monodisperse, PVP-coated AgNP (70 nm) showing little agglomeration in aqueous suspension were instilled intratracheally. After 24 hours, the lungs were lavaged, and lactate dehydrogenase (LDH), total protein, and cytokine levels as well as total and differential cell counts were measured in the bronchoalveolar lavage fluid (BALF). Instillation of 50 µg PVP-AgNP did not result in elevated LDH, total protein, or cytokine levels in BALF compared to the control, whereas instillation of 250 µg PVP-AgNP caused a significant increase in LDH (1.9-fold) and total protein (1.3-fold) levels as well as in neutrophil numbers (60-fold) of BALF. Furthermore, while there was no change in BALF cytokine levels after the instillation of 50 µg PVP-AgNP, instillation of 250 µg PVP-AgNP resulted in significantly increased levels of seven out of eleven measured cytokines. These finding suggest that exposure to inhaled AgNP can induce moderate pulmonary toxicity, but only at rather high concentrations.

## Introduction

Silver nanoparticles (AgNP) are among the most promising nanomaterials, and their usage in medical applications and consumer products is growing rapidly [1]. The antimicrobial properties of AgNP render them useful as a component in wound dressings or as coatings for catheters [2–5]. In addition, they are used in deodorants or applied in textiles as a protection against odor [6–7].

With regard to the use in hygiene and health care spray products, AgNP had become present in everyday life. In case of such aerosolized AgNP, the lungs with their large surface area are the first organs that come into contact with inhaled AgNP [8–9].

Inhaled particles with a diameter of less than 100 nm mainly deposit in the alveolar region [10–12]. Once deposited there, nanoparticles are found to interact with the epithelial lining fluid including pulmonary surfactant, lung macrophages and epithelial cells [13–15]. Depending on their physico-chemical properties, a small portion of the inhaled nanomaterials may even be able to cross the air-blood-barrier (ABB), towards circulation, and accumulate in secondary organs [16–17].

Some in vitro studies have demonstrated toxic effects of AgNP on lung cells: In vitro incubation of a rat alveolar macrophage cell line with AgNP induced a concentration- as well as a size-dependent decrease in cell viability. In addition, a proinflammatory response was shown by increased levels of tumor necrosis factor-α (TNF-α), macrophage inflammatory protein-2 (MIP-2), and interleukin-1β (IL-1β) [18]. Furthermore, AgNP caused damage to mitochondria and an increased production of reactive oxygen species (ROS) in human lung fibroblasts in a dose-dependent manner [19]. In contrast, very little is known about the cytotoxic and proinflammatory effects of AgNP in the respiratory system in vivo [20]. Intratracheal instillation of slightly agglomerated AgNP in mice resulted in progressively increased levels of IL-1, TNF-α, and IL-6 by day 28 after a single instillation [21]. In another mouse study, however, only minimal lung toxicity and inflammation were found after subacute inhalation of AgNP [22]. In addition, there are only two in vivo studies dealing with adverse pulmonary effects of AgNP in rats.

In these studies, the subchronic inhalation of AgNP caused lung function changes as well as chronic alveolar inflammation and small granulomatous lesions [23–24]. In two other studies from the same group, however, acute and subchronic inhalation of AgNP at lower doses and shorter inhalation times did not cause adverse health effects in rats as measured by lung function, hematology, and body weight chances [25–26].

The mechanisms of toxicity are proposed to be oxidative stress, DNA damage, and the modulation of cytokine production [20]. In addition, Liu et al. showed that AgNP undergo profound chemical transformations in biological environments that can affect bioavailability and toxicity. In case of argyria, silver deposits in the skin are not translocated engineered AgNP, but rather secondary particles formed of silver metabolites resulting from partial AgNP dissolution and subsequent metabolization of silver ions. Thus, the dissolution of AgNP and release of silver ions as well as the subsequent biochemical transformations are an important issue in AgNP toxicity [27]. However, most of the information available about the mechanisms of AgNP toxicity has been derived from in vitro studies. The aim of the current study was to assess the adverse health effects of AgNP in vivo, more specifically, after the intratracheal instillation in rats, with a focus on cytotoxicity and cytokine induction. Therefore, monodisperse polyvinylpyrrolidone (PVP)-coated AgNP (70 nm mean diameter) were instilled intratracheally into healthy rats, and cytotoxic and proinflammatory effects were determined by measuring lactate dehydrogenase (LDH), protein, and cytokine levels as well as total and differential cell counts in bronchoalveolar lavage fluid (BALF).

## Results

### Particle characterization

**Figure 1:**
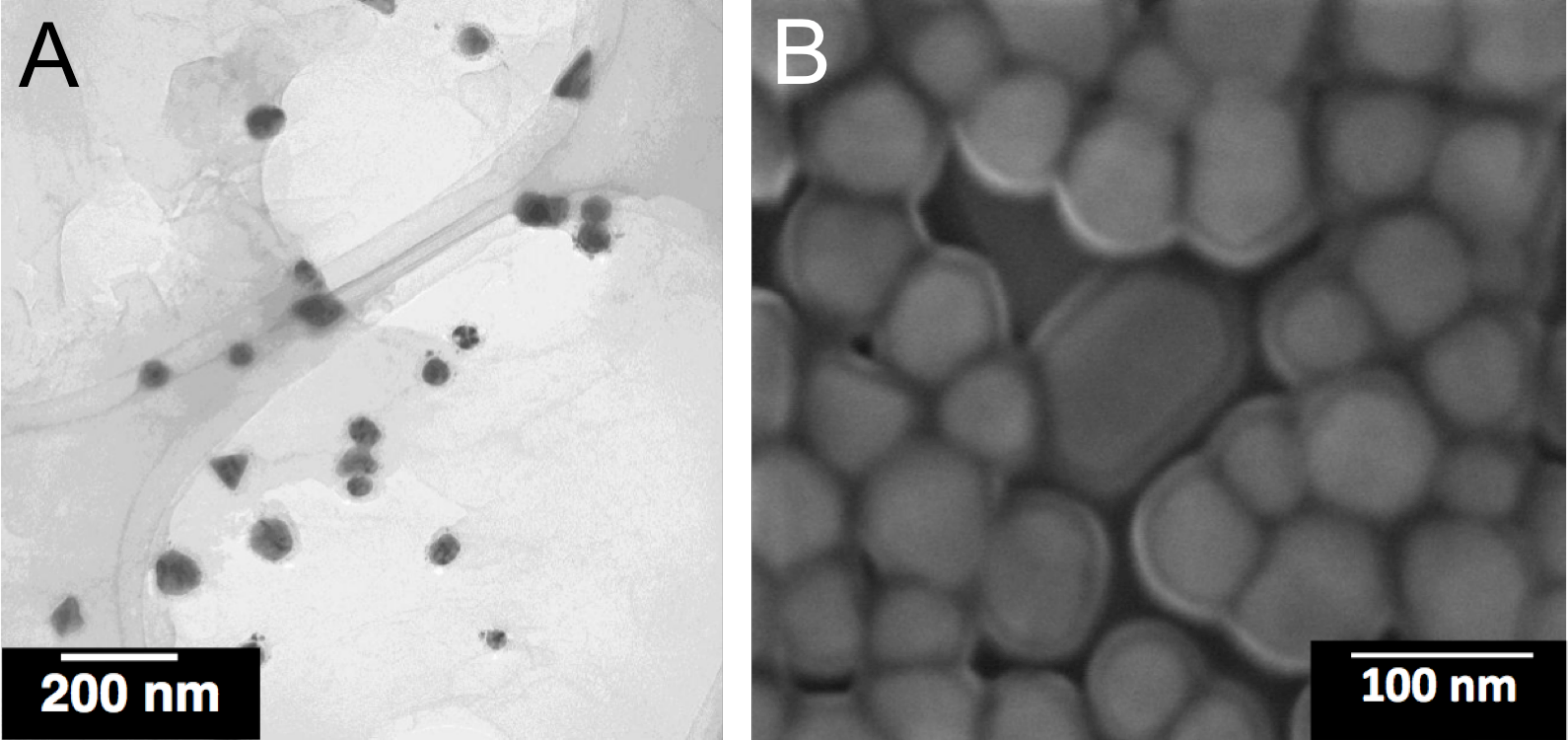
Representative transmission (A) and scanning electron (B) images of PVP-coated AgNP.

**Figure 2:**
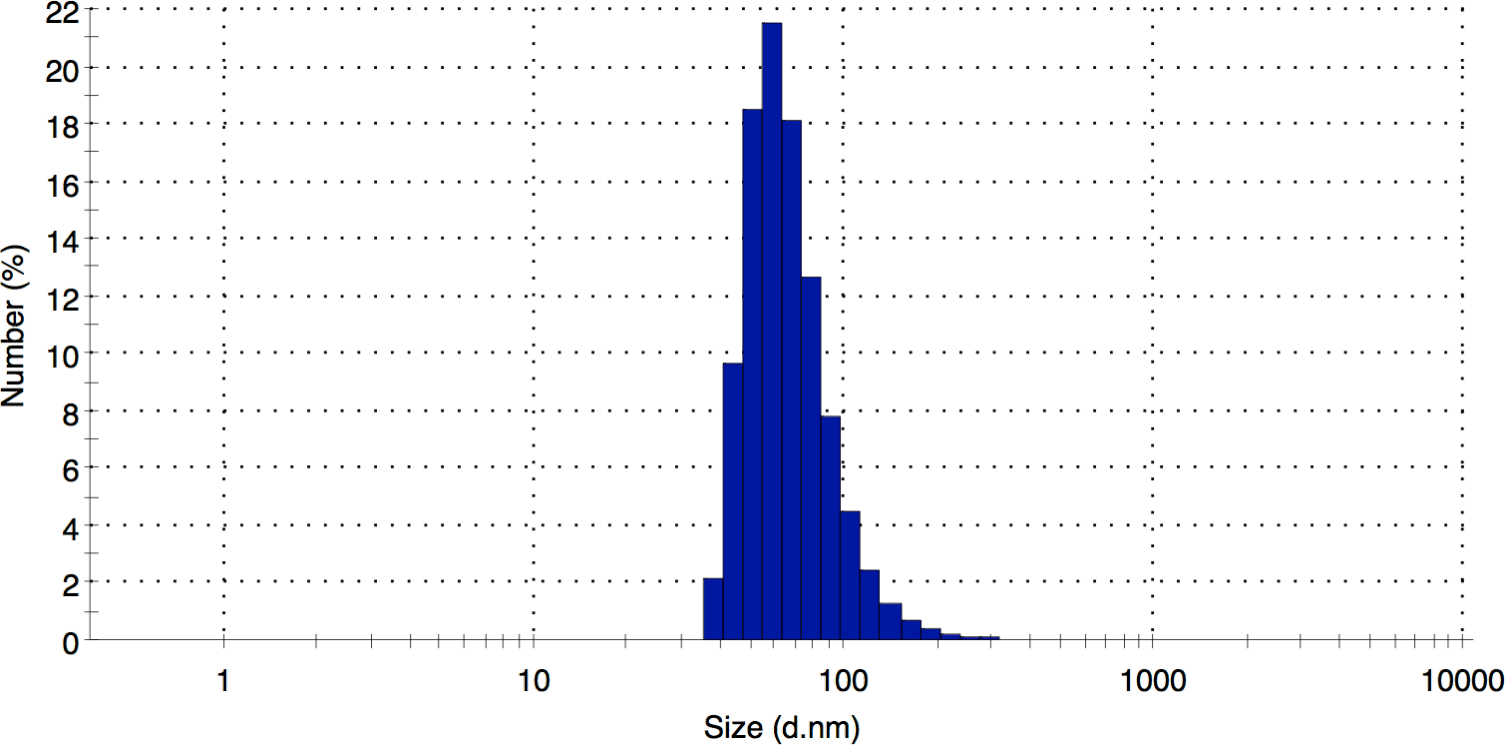
Particle number distribution of PVP-AgNP by dynamic light scattering (Nano Zetasizer ZS, Malvern, Herrenberg, Germany).

**Figure 3:**
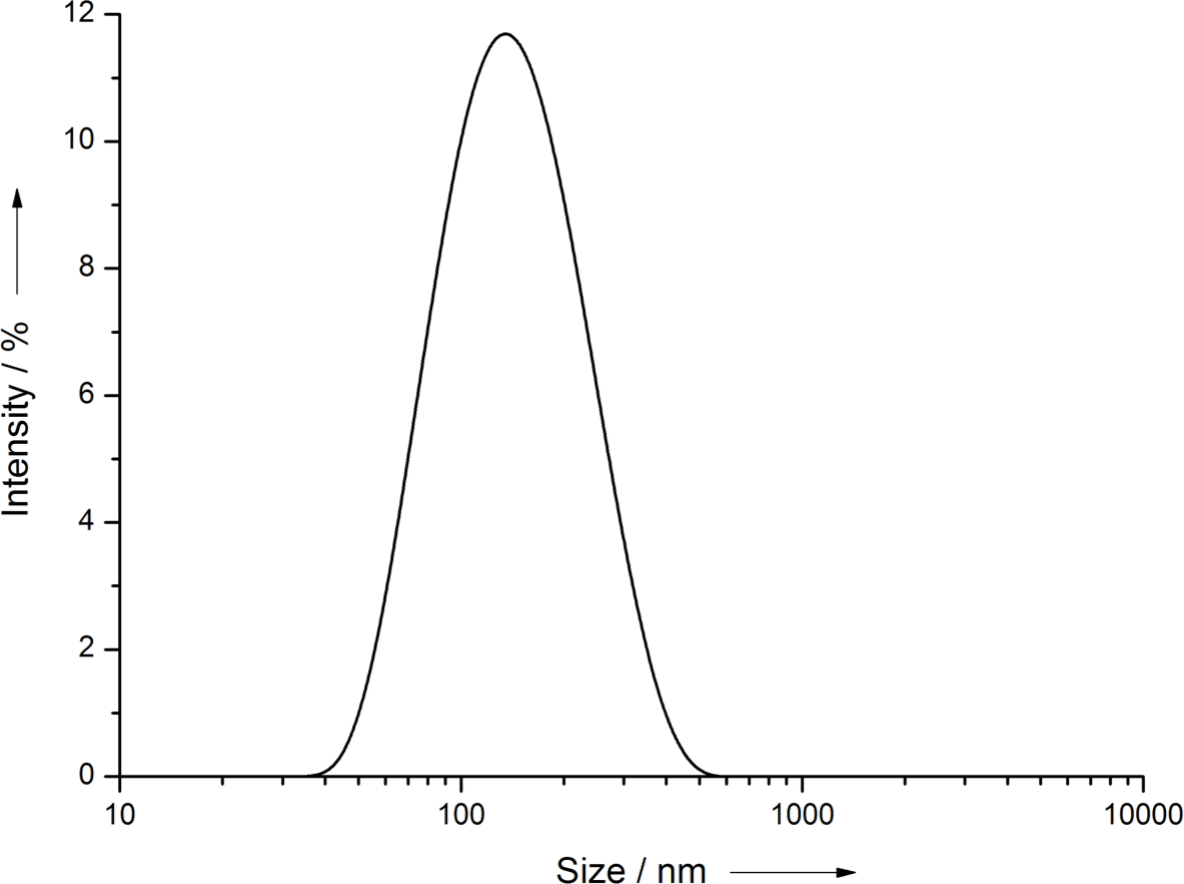
Particle intensity distribution of PVP-AgNP by dynamic light scattering (Nano Zetasizer ZS, Malvern, Herrenberg, Germany).

The mean diameter of the PVP-coated AgNP was 70 nm as measured by electron microscopy (Figure 1). The *z*-average was 123 nm and the polydispersity index 0.18 as determined by dynamic light scattering. The size distribution by number and the particle intensity distribution are shown in Figure 2 and Figure 3.

### LDH and total protein levels in BALF

**Figure 4:**
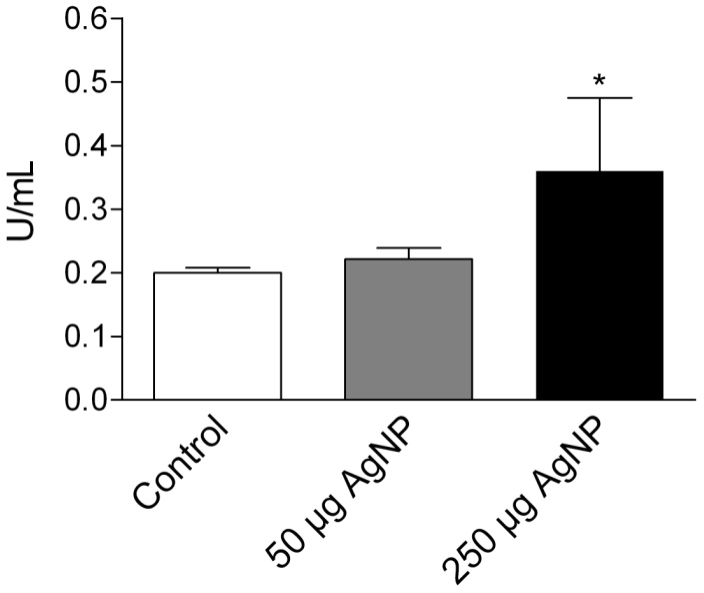
LDH levels in BALF 24 hours after intratracheal instillation of PVP-AgNP. Values are mean ± SEM; *n* = 5 for each treatment group; *p < 0.05 vs control.

The stable enzyme LDH is localized in the cytoplasm of cells. Consequently, the destruction of cell membranes results in an increased LDH release from cells. This release due to the loss of membrane integrity is, therefore, a marker of cytotoxic effects. As shown in Figure 4, there was no significant increase in LDH levels in BALF after instillation of 50 µg PVP-AgNP into the rat’s lung. However, instillation of 250 µg PVP-AgNP caused a significant increase in LDH release as compared to controls (0.19 ± 0.01 U/mL to 0.36 ± 0.05 U/mL).

**Figure 5:**
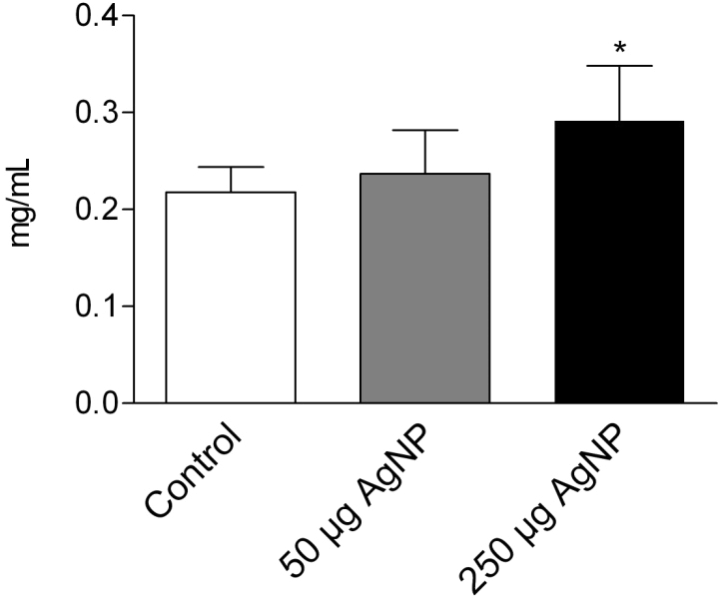
Total protein levels in BALF 24 hours after the intratracheal instillation of PVP-AgNP. Values are mean ± SEM; *n* = 5 for each treatment group; *p < 0.05 vs control.

Lung injury allows proteins to overcome the ABB, resulting in increased BALF protein concentrations. Similar to the results from the LDH measurements, there was no change in BALF protein levels after the instillation of 50 µg of PVP-AgNP when compared to the controls. However, the instillation of 250 µg PVP-AgNP caused a low but significant increase in BALF protein levels (Figure 5).

### Cytokine levels in BALF

**Figure 6:**
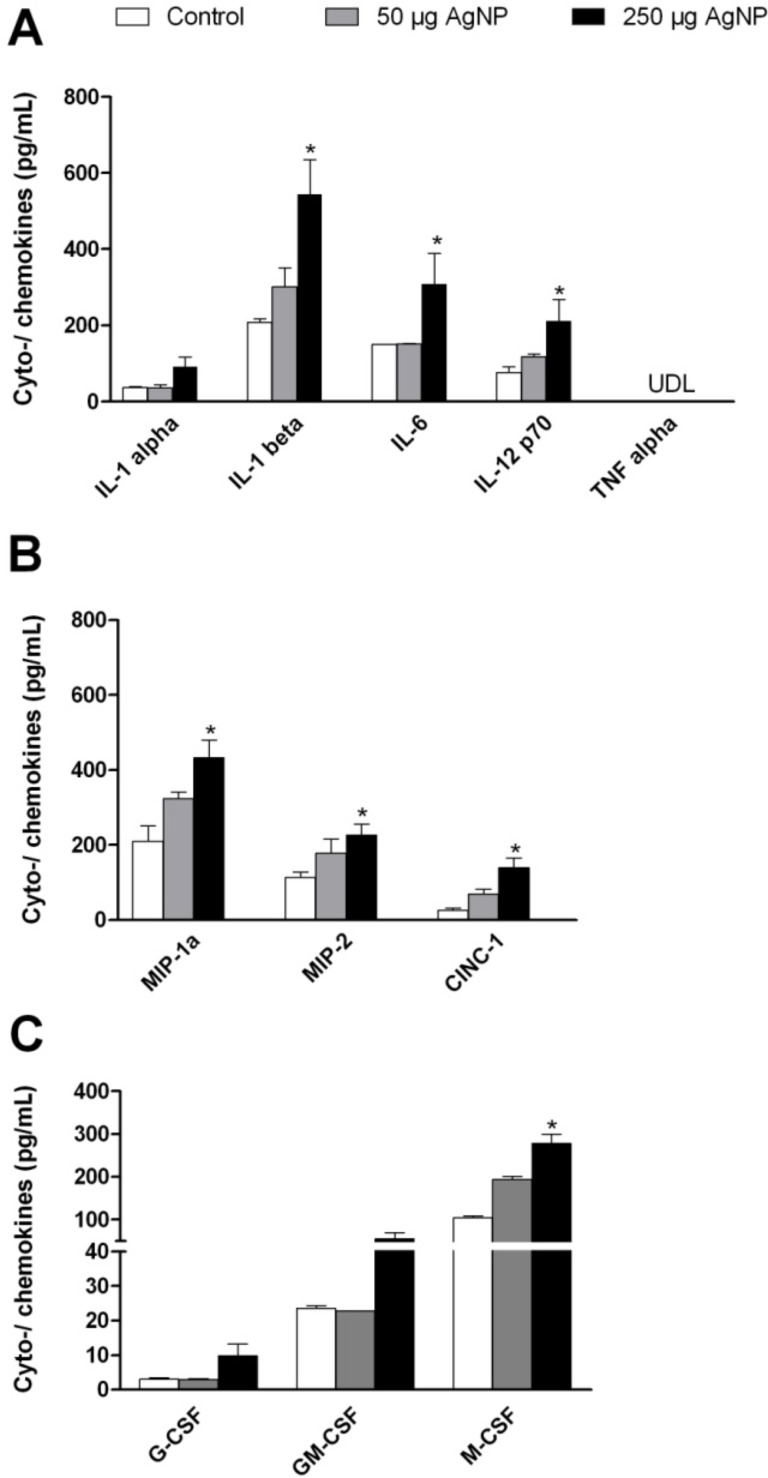
Cytokine levels in BALF 24 hours after the intratracheal instillation of PVP-AgNP (A: proinflammatory cytokines; B: chemokines; C: colony-stimulating factors). For the lower limits of detection see “Experimental”. Values are mean ± SEM; *n* = 5 for each treatment group; *p < 0.05 vs control. UDL = under detection limit.

To assess the proinflammatory effects of PVP-AgNP, we determined the BALF levels of several cytokines and chemokines after intratracheal instillation. Macrophage activators such as IL-1α, IL-1β, IL-6, and IL-12p70 act as proinflammatory cytokines. Figure 6A shows that the instillation of 250 µg PVP-AgNP caused an increase in the BALF levels of all four cytokines as compared to controls that was significant for IL-1β, IL-6, and IL-12p70. TNF-α, another proinflammatory cytokine, was not detectable in BALF. Similarly, significantly elevated levels of CINC-1 as well as of the macrophage inflammatory proteins 1-α and 2 (MIP-1α, MIP-2) were found after the instillation of 250 µg (Figure 6B). Moreover, the instillation of 250 µg PVP-AgNP resulted also in a significantly increased level of the macrophage-/colony stimulating factor (M-CSF) as compared to controls (Figure 6C).

### Cell counts in BALF

Lungs were lavaged 24 hours after the instillation of 50 or 250 µg PVP-AgNP and total as well as differential cell counts were determined as described before. The pulmonary influx of neutrophils was considered to be a marker of inflammation.

**Figure 7:**
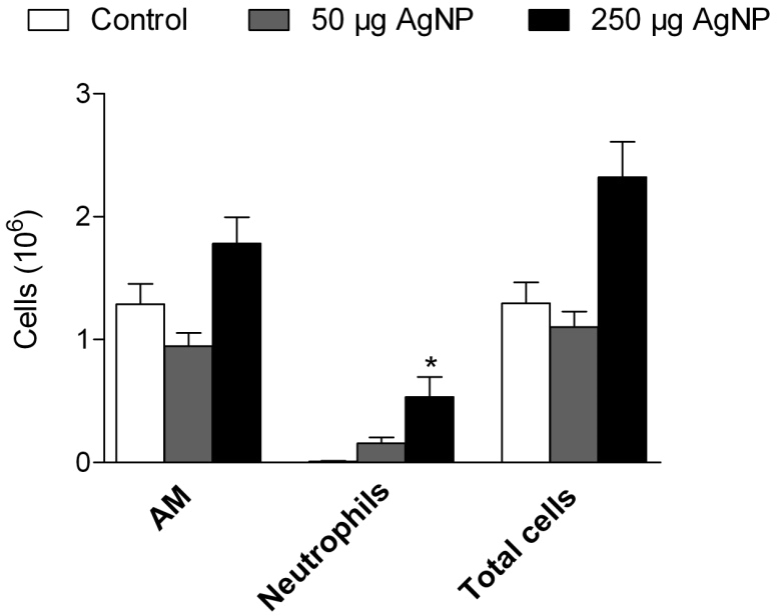
Cell counts in BALF 24 hours after the instillation of PVP-AgNP. AM: Alveolar macrophages. Values are mean ± SEM; *n* = 5 for each treatment group; *p < 0.05 vs control.

**Figure 8:**
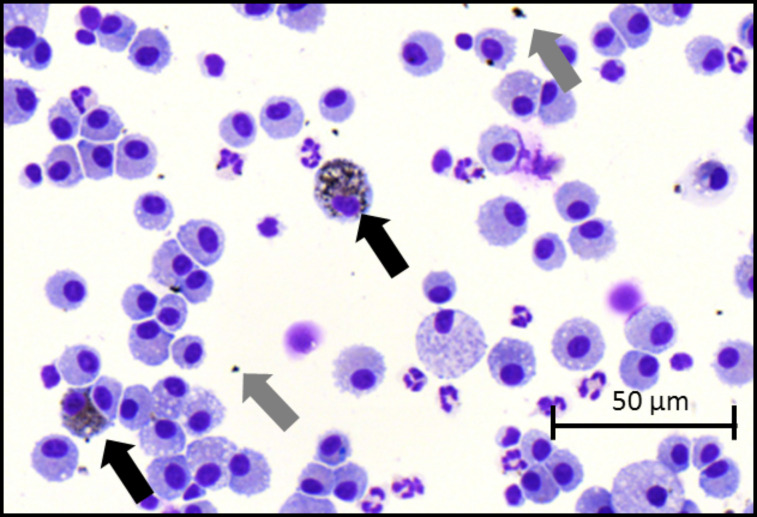
Representative BAL cell image after the intratracheal instillation of 250 µg PVP-AgNP. Gray arrows indicate free particles; black arrows indicate alveolar macrophages with internalized PVP-AgNP.

While there was no significant difference in total cell counts between control rats and rats exposed to 50 µg PVP-AgNP, the instillation of 250 µg PVP-AgNP resulted in a 2-fold increase in BALF total cell counts. Furthermore, while the instillation of 50 µg PVP-AgNP caused a slight increase (17-fold) in the number of neutrophils that did not reach statistical significance, the instillation of 250 µg PVP-AgNP produced a significant influx (60-fold) of neutrophils into the lungs (Figure 7). Representative images of BAL cells show the presence of PVP-AgNP both in alveolar macrophages and free particles/agglomerates in BALF after the instillation of 250 µg PVP-AgNP (Figure 8).

## Discussion

According to the Woodrow–Wilson-Center database of nanotechnology-based products [28], silver is one of the most frequently used nanomaterials for consumer products. Due to the use of AgNP as aerosols in healthcare and hygiene spray products, the lungs are considered to be the main portal of entry for AgNP into the human body [29]. With regard to the lack of knowledge of the in vivo pulmonary toxicity of AgNP, the aim of the current study was to assess the adverse health effects of AgNP after the intratracheal instillation in rats, with a focus on cytotoxicity and cytokine induction. Here, we demonstrate that the intratracheal instillation of 250 µg, but not of 50 µg, of monodisperse PVP-coated 70 nm AgNP in rats caused cytotoxic and inflammatory responses of the lungs, as shown by elevated BALF LDH, protein, and cytokine levels as well as neutrophil numbers.

These findings are in line with previous mouse studies: The intratracheal instillation resulted in increased BALF levels of IL-1, TNF-α, and IL-6 [21] and the inhalation caused increased BALF levels of IL-12(p40) and keratinocyte chemoattractant (KC) [22]. In contrast to the present study, cytotoxicity was not assessed in the instillation study of Park and co-workers [21] nor were found elevated BALF LHD and protein levels in the inhalation study of Stebounova et al. [22]*.* In contrast to these mouse studies, information about cytotoxic and inflammatory effects after the inhalation of AgNP in rats is very scarce. Two studies demonstrated adverse pulmonary effects such as lung function changes as well as chronic alveolar inflammation and small granulomatous lesions in histopathological examinations [23–24]. But in contrast to our study, BALF cytokine levels were not determined. In addition, the results after the inhalation of AgNP in rats are diverging. In two other studies from the same group, acute and subchronic inhalation of AgNP at lower doses and shorter inhalation times did not cause adverse health effects in rats as measured by lung function, hematology, and body weight chances [25–26]. The lower doses used and the shorter inhalation times in comparison to the studies where adverse health effects occurred might be the reasons for the diverging results. Unfortunately, direct pulmonary responses such as LDH, protein, and cytokine levels in BALF were not assessed in these studies.

Several in vitro studies dealt with the mechanism of cytokine induction after AgNP exposure. Incubation of human mesenchymal stem cells and of peripheral blood monocytes with the same PVP-AgNP that were used in the present study induced a concentration-dependent uptake of particles into the cells and a subsequent release of the proinflammatory cytokines IL-6 and IL-8 [30–31]. Moreover, Greulich et al. described the internalization of AgNP to be a clathrin-mediated process [31]. The clathrin-mediated process of internalization after incubation of human macrophages with AgNP was stated to be a trigger for immune responses [32] and responsible for the production of IL-8 [33]. AshaRani and co-workers demonstrated the involvement of the NFκB and MAP kinase pathways after exposure of human lung cells (IMR-90) to AgNP. Consequently, the activation of these pathways resulted in the transcription of genes involved in proliferation and inflammatory responses. Likewise, an up-regulation of IL-6, IL-8, M-CSF and MIP-1β following AgNP exposure was demonstrated [34]. Since comparable proinflammatory responses were found in the present study, we suggest that the inflammatory reaction induced by the intratracheal instillation of 250 µg AgNP was caused by similar mechanisms as those described previously in the in vitro studies.

In the present study, adverse pulmonary effects occurred only after the instillation of 250 µg PVP-AgNP. As the intratracheal instillation of 50 µg silver nanoparticles did not result in cytotoxic or inflammatory effects, the rationale to use an additional dose of 250 µg (1 mg/kg body weight) was to induce potential toxic effects but not death or severe suffering to the animal. To estimate whether the toxicity is driven by AgNP doses that are so high that cells cannot deal with, we assessed cellular doses. Although the intratracheal instillation will not lead to a uniform distribution in the lungs, for simplicity we assume a nearly uniform distribution of PVP-AgNP in the present study. As Stone and co-workers [35] determined the total number of cells in the alveolar region to be 8.9·10^8^ in the rat’s lung, the average concentration after the intratracheal instillation of 250 µg AgNP is estimated to be 0.28 pg AgNP/cell corresponding to 150 AgNP per cell in the alveolar region. When presuming that all AgNP would have been phagocytized by the 27·10^6^ alveolar macrophages in the rat lungs, each of the macrophages will have received an average dose of 9.6 pg AgNP corresponding to about 5.3·10^3^ AgNP/cell and about 1 µm^3^ AgNP volume per cell while the volume of a rat alveolar macrophage of 12 µm is 900 µm^3^. Tran and co-workers found that alveolar macrophages are able to deal with a particle volume of approximately 60 µm^3^ per cell [36]. At higher particle volumes the normal clearance capacity of alveolar macrophages is affected. These calculations suggest that the cytotoxic and inflammatory effects observed in the present study were not due to a high AgNP dose per epithelial cell or alveolar macrophage, but are in good agreement with the toxicity mechanisms of dissolved Ag ions described above.

Another crucial point in the discussion about the toxicity of AgNP is the release of silver ions. Kittler and co-workers noted that the rate of the dissolution of AgNP depends on the surface functionalization, concentration and temperature [37]. They found an increasing toxicity to human mesenchymal stem cells during the storage of AgNP solutions, explained by the increasing release of silver ions over time. The authors emphasized that the dissolution behavior of AgNP in biological media would be different, as their studies were carried out in ultrapure water. In the present study we used 70 nm, PVP-coated AgNP. Since the dissolution rate is proportional to the specific surface area of NP [38] their dissolution rate is 3.5 times lower than those of 20 nm AgNP used in the toxicity studies funded by the EC [28] and mediated by OECD. In comparison to other stabilizing agents, PVP-AgNP were found to be the most stable in OECD media [39] and PVP itself minimized the dissolution of silver ions [40]. Recent studies reported that PVP itself does not exert any toxic effect [30,41]. In other studies, however, toxic effects of PVP-coated AgNP were found both in vivo as well as in vitro [42–44], indicating that PVP does not mask the toxicity of AgNP.

## Conclusion

In conclusion, the intratracheal instillation of 250 µg, but not of 50 µg, of 70 nm monodisperse PVP-coated AgNP caused cytotoxic and inflammatory responses in lungs of healthy, adult female rats, as shown by elevated BALF LDH, protein, and cytokine levels as well as neutrophil numbers. These findings suggest that exposure to inhaled AgNP can induce a moderate pulmonary toxicity, but only at rather high concentrations.

## Experimental

### Animals

Female Wistar–Kyoto rats (WKY/Kyo@Rj rats, Janvier, Le Genest Saint Isle, France), 8–10 weeks of age (approx. 250 g body weight), were housed in pairs in relatively humidity- and temperature-controlled ventilated cages (VentiRack Bioscrene TM, Biozone, Margate, UK) on a 12-hour day/night cycle. Rodent diet and water were provided ad libitum. All experiments were conducted under German federal guidelines for the use and care of laboratory animals and were approved by the Regierung von Oberbayern (Government of District of Upper Bavaria, Approval No.55.2-1-54-2531-26-10) and by the Institutional Animal Care and Use Committee of the Helmholtz Center Munich.

### PVP-coated Ag nanoparticles (PVP-AgNP)

PVP-AgNP were prepared by reduction with glucose in the presence of PVP as described before [37,45]. The silver nanoparticles were dispersed in ultrapure, degassed water. The final silver concentration was determined by atomic absorption spectroscopy (AAS). The typical yield of Ag was about 5%. Unreacted silver and synthesis byproducts were removed by multiple ultracentrifugation, followed by redispersion under ultrasonication (ultrasound bath). Scanning electron microscopy (SEM) was performed with an ESEM Quanta 400 instrument with gold/palladium-sputtered samples. Transmission electron microscopic (TEM) was carried out with a CM200 FEG-Instrument (Philips) with a Supertwin lens, operated at an accelerating voltage of 200 keV. The samples were ultrasonically dispersed (ultrasound bath) in ethanol and then transferred to holey carbon-coated copper grids. Immediately prior to intratracheal instillation, the particle size distribution and the polydispersity index were measured by dynamic light scattering with a Malvern Zetasizer Nano ZS (Malvern Instruments GmbH, Herrenberg, Germany).

### PVP-AgNP administration

Non-fasted animals were intratracheally instilled with 80 µL of PVP-AgNP suspension (containing either 50 µg or 250 µg AgNP). For this purpose, rats were anesthetized by MMF anesthesia (150 µg/kg medetomidin, 2 mg/kg midazolam, 5 µg/kg fentanyl). The anesthetized rat was fixed with its incisors to a rubber band on a board in supine position at an angle of 60° to the lab bench. A flexible cannula (2.7 × 50 mm, B. Braun, Melsungen, Germany) was placed under visual control into the upper third of the trachea. For the identification of misplacement into the esophagus, respiration through the cannula was monitored with a pneumotachograph connected to the cannula. The PVP-AgNP suspension was gently instilled during inspiration followed by 300–400 µL of air by using a 1 mL insulin syringe. After instillation, the MMF anesthesia was antagonized by injection of a mixture of atipamezol (0.75 mg/kg), flumazenil (200 µg/kg), and naloxon (120 µg/kg) according to the rat’s weight. Rats that did not receive PVP-AgNP served as controls.

### Bronchoalveolar lavage (BAL)

The rats were anesthetized (5% isoflurane inhalation) 24 hours after instillation of the AgNP suspension, and euthanized by exsanguination via the abdominal aorta. A flexible cannula (2.7 × 50 mm, B. Braun, Melsungen, Germany) was placed into the upper third of the trachea. With a syringe connected to the cannula, 5 mL PBS were gently instilled into the lungs, while the thorax was massaged carefully. Then, the lavage fluid was gently aspirated and the procedure was replicated four times. The lavages were pooled and centrifuged at 400 g for 30 min at 4 °C. The cell free supernatant (BALF) was used for biochemical measurements of LDH, total protein, and cytokine levels.

### Determination of protein and LDH levels in BALF

The total protein content in the BALF supernatant was determined spectrophotometrically at a wavelength of 560 nm with the Pierce^®^ BCA Protein Kit Assay (Thermo scientific, Rockford, USA). LDH activity was determined spectrophotometrically at a wavelength of 490 nm with the Cytotoxicity Detection KitPLUS (LDH; Roche Diagnostics, Mannheim, Germany). Both assays were performed according to the manufacturers’ instructions.

### Quantification of TNF-α and CINC-1 levels in BALF by ELISA

TNF-α and cytokine-induced neutrophil chemoattractant-1 (CINC-1) were measured in BALF supernatant by using a commercially available enzyme-linked immunosorbent assay kit (ELISA DuoSet, R&D, Wiesbaden-Nordenstadt, Germany). The ELISA was performed according to the manufacturer’s specifications. Lower limits of detection were 30 pg/mL for TNF-α and 15 pg/mL for CINC-1. Absorbance was determined at a wavelength of 450 nm.

### Measurement of cytokines and chemokines in BALF by a multiplex bead array assay

The levels of nine relevant cytokines and chemokines, including granulocyte-colony stimulating factor (G-CSF), granulocyte macrophage-colony stimulating factor (GM-CSF), macrophage-colony stimulating factor (M-CSF), interleukin (IL)-1α, IL-1β, IL-6, IL-12p70, macrophage inflammatory protein (MIP)-1α, and MIP-2, were determined in BALF supernatant. The cytokine multiplex bead array assay kit was purchased from BioRad (Bioplex cytokine assay, Munich, Germany). The kit was used according to the manufacturer’s specifications. Lower limits of detection were 16 pg/mL for G-CSF, 22 pg/mL for GM-CSF, 95 pg/mL for M-CSF, 35 pg/mL for IL-1α, 116 pg/mL for IL-1β, 150 pg/mL for IL-6, 67 pg/mL for IL-12p70, 96 pg/mL for MIP-1α, and 72 pg/mL for MIP-2.

### Cytological analysis of BAL

The remaining cell pellet was resuspended in 1 mL RPMI 1640 medium (BioChrome, Berlin, Germany) supplemented with 10% fetal calf serum (Seromed, Berlin, Germany). Cell counts were measured by a CASY cell counter system (Schärfe System GmbH, Reutlingen, Germany). Cytospins were prepared by cytocentrifugation at 35*g* for 6 min and stained with May–Grünwald–Giemsa. 200 cells per each slide were differentially counted under a light microscope.

### Statistical analysis

Data in the figures are given as mean ± SEM. Statistical analysis was performed by one-way analysis of variance (ANOVA) on ranks followed by a post hoc Tukey’s multiple comparison test or Dunn’s test (Software: SigmaStat for Windows, Jandel Scientific, Erkrath, Germany). Differences between rats exposed to PVP-AgNP and controls were considered statistically significant at p < 0.05.
